# Molecular and morphological approach to study the innexin gap junctions in *Rhynchosciara americana*

**DOI:** 10.1098/rsob.210224

**Published:** 2021-11-10

**Authors:** Jorge Henrique Neves, Paula Rezende-Teixeira, Natalia Bazan Palomino, Glaucia Maria Machado-Santelli

**Affiliations:** Institute of Biomedical Sciences, University of São Paulo, Avenida Professor Lineu Prestes, 1524 – sala 307, São Paulo, SP, Brazil

**Keywords:** gap junction, innexin, *Rhynchosciara americana*, biological development

## Abstract

Gap junctions mediate communication between adjacent cells and are fundamental to the development and homeostasis in multicellular organisms. In invertebrates, gap junctions are formed by transmembrane proteins called innexins. Gap junctions allow the passage of small molecules through an intercellular channel, between a cell and another adjacent cell. The dipteran *Rhynchosciara americana* has contributed to studying the biology of invertebrates and the study of the interaction and regulation of genes during biological development. Therefore, this paper aimed to study the *R. americana* innexin-2 by molecular characterization, analysis of the expression profile and cellular localization. The molecular characterization results confirm that the message is from a gap junction protein and analysis of the expression and cellular localization profile shows that innexin-2 can participate in many physiological processes during the development of *R. americana*.

## Graphical abstract

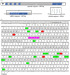

## 1. Introduction

*Rhynchosciara americana* is a dipteran belonging to the Sciaridae family, which, due to its characteristics, has contributed to the knowledge of the cellular and molecular biology of insects, such as the identification of DNA puffs, by Breuer & Pavan in 1955 [1] and Machado-Santelli & Basile in 1975 [2]. The characteristics of the species *R. americana* are a long-life cycle, synchronous development among sibling larvae of the same sex [3]. Furthermore, the polytene chromosomes of this sciarid are large with favourable morphology for cytological preparations, polytene chromosomes in different tissues and the DNA amplification phenomenon during larval development give rise to DNA puffs and RNA puffs [1].

Communication between the cytoplasm of one cell with an adjacent cell is essential. Intercellular junctions play a fundamental role in several processes throughout the development of insects, such as during oogenesis, embryogenesis and nervous system development [4]. In animals, the communicating junctions are formed by protein subunits that enable intercellular communication, allowing ions and small molecules to pass through juxtaposed channels of adjacent cells [4–6]. Protein subunits are responsible for the perfect coupling between a hemichannel and an adjacent one, forming an intercellular channel [7]. Thus, a hemichannel consists of a ring of multimeric proteins, where each protein has four alpha-helix transmembrane domains, two extracellular loops, one intracellular loop, and the amino-terminal (AT) and carboxy-terminal (CT) ends are located intracellularly [8,9].

In vertebrates, the cell junctions known as gap junctions are formed by connexins and pannexins, and the innexins form gap junctions in invertebrates [10,11]. Studies of electron cryo-microscopy and three-dimensional reconstruction revealed that innexin-6 has a larger dimension than connexin-26, forming hexadodecameric channels [12,13]. The junctions that form the hexadodecameric channels are formed by two octameric channels (eight subunits), forming an intercellular channel of 16 subunits. Although connexins and innexins have functional homology, they have no homology in the amino acid sequence [4,14]. However, members of the pannexin family present in vertebrates have homology with the amino acid sequence of innexins, but the alignment of the sequences does not show high identity [15,16].

In invertebrates, gap junctions were first identified by Phelan *et al*. [14], who showed that the Shaking-B protein could form intercellular channels. In invertebrates, 21 genes from the innexin family were found in the nematode *C. elegans* and medicinal leeches [17,18]. In comparison, there is less diversity of innexin genes in arthropods. Genomic studies in *Drosophila melanogaster* identified eight genes from the innexins family with several different isoforms [4,11,19]. Studies carried out on *Bombyx mori* identified three innexins [20,21]. In *Aedes aegypti*, six genes encoding innexins were found, and another six genes were found in the stomatogastric ganglion of *Cancer borealis* [22,23]. In *Homarus americanus*, 13 gene products from the innexin family were found, six known genes (Homam-Inx1–4 and Homam-Inx6–7) and seven new putative innexin genes (Homam-Inx8–14) [24].

Innexins have an essential role in the development of insects and other arthropods. In *D. melanogaster*, Dm-Inx1 participates in the development of the nervous system [25]. Dm-Inx1 and Dm-Inx8 work together to form the photoreceptors of the retina [26]. Studies in *D. melanogaster* show that Dm-Inx3 participates in the dorsal closure of the embryo [27]. In *Anopheles gambiae*, disturbances in the expression of Ag-Inx4 result in sterile males, and Ag-Inx7 is essential for the development of the embryonic nervous system [28,29]. In *Tribolium castaneum*, Tc-Inx7 participates in the blastoderm cellularization process [30].

Innexin-2 is one of the most studied innexins in insects. Dm-Inx2 participates in epithelial tissue embryonic morphogenesis [31–34]. Studies show that the junctions formed by Dm-inx2 mediate intercellular calcium transfer during healing and the passage of GDPL-Fucose in the imaginal disk of the wings [35,36]. Dm-Inx2 also participates in developing the eyes and the central nervous system [37,38]. Studies carried out in *Drosophila* show that Inx2 acts on calcium transportation between follicular cells (FC) during oogenesis [39]. Dm-Inx2 and Dm-Inx3 participate in the transmission of bioelectric signals during oogenesis [40]. In the culture of contractile cells of the ventral diverticulum of *A. aegypti*, Inx2 and Inx7 were the most expressed among innexins, with Inx2 being the most expressed [41]. Studies carried out in mosquitoes (*Aedes albopictus*) show the expression of Inx2 in the contact regions of the cells with other adjacent cells and on the free surface of the cells [42]. In *Scylla paramamosain*, Sp-Inx2 has greater expression in cells of the immune system [43]. Inx2 was expressed in striated muscle cells of an American cockroach (*Periplaneta americana*), participating in muscle contractile signalling [44].

Gap junctions have been observed in several organs and tissues performing the function of cell–cell communication; however, innexin proteins also seem to be important for developing organs and tissues. The main objective of this work was to study the innexin-2 of *Rhynchosciara americana* (Ra-Inx2) through molecular and morphological techniques. In addition to associate the role of the innexin-2 in the formation of gap junctions during *Rhynchosciara americana* development evaluating: (i) the nucleotide sequence and the putative protein identifying characteristics of gap junction proteins through bioinformatics analysis, (ii) the mRNA expression profile in germline and somatic lineages and (iii) cellular localization of the protein in *R. americana* organs and gene localization of the Ra-Inx2 gene in polytene chromosomes.

## 2. Methods

### 2.1. Animals

*Rhynchosciara americana* larvae were collected in the Ubatuba region, state of São Paulo, Brazil, and grown in the laboratory, using the conditions established by Lara *et al*. [45], with modifications.

### 2.2. Nucleic acid extraction and qPCR

Nucleic acids were extracted using TMD solution (25 mM Tris pH 7.5; 20 mM EDTA pH 8.0; and 20 mM NaCl), 10% SDS and 200 mg ml^−1^ Proteinase K (Sigma Aldrich). The reaction was incubated for approximately 60 min at 50°C. Then, 1 V phenol : chloroform : isoamyl alcohol (25 : 24 : 1) was added, mixed by inversion and then centrifuged for 5 min at 14 000 rpm. The aqueous phase was transferred to a sterile tube for precipitation and 2.5 V of absolute ethanol and 0.1 V of 3 M sodium acetate pH 5.0 was added. The DNA was resuspended in TE pH 8.0 (10 mM Tris HCl, 1.0 mM EDTA) and treated with RNase, while the RNA was resuspended in H_2_O RNase Free and treated with DNase (Kit: DNase 1, Amplification Grade, SigmaAldrich) after the nucleic acids were quantified in the NanoDrop ND1000 Spectrophotometer (Thermo Scientific).

The cDNA synthesis was performed using the Improm II Kit - Reverse Transcription System (Promega). The reaction was processed in the Veriti Thermal Cycler (Applied Biosystems). The analysis of the expression profile (quantitative PCR) was done using the GoTaq^®^ qPCR Master Mix (Promega), and the reactions were carried out into Corbett Research Rotor Gene 6000 real-time cycler (Qiagen, Hilden, Germany) under the following conditions: 94°C for 5 min, 40 cycles (94°C for 20 s, 53°C for 20 s and 72°C for 30 s), following melting. The youngest period was used as a calibrator and the results obtained were analysed in the REST 2005 program. Primers used qRaInx2_Left (5′-ACGTGTGCTGCGAAGTTATG-3′) and qRaInx2_Right (5′-AGCGTAAGCAAGCAGAGAGC-3′).

### 2.3. PCR amplification and sequencing

Specific primers for each Ra-Inx2 region are listed in table 1. PCR reactions were performed on a Veriti Thermal Cycler (Applied Biosystems), the generated amplicons were cloned using the pGEM-T easy kit (Promega), and after sequencing.

**Table 1.** Primer sequences used in PCR amplification and quantitative PCR.

primers	sequence	TM (°C)
Inx2_5′	5′-TTATGACATCGGCGTTCAGA-3′	53.9
Inx2_InSitu_Left	5′-CGATGCATTATGTTGGGCGA-3′	55.9
Inx2_InSitu_Right	5′-AGGTGGAAAATGTGGGACCT-3′	56.4
5′_Inx2_Left	5′-ACTTGATGAGCCGGACTAAA-3′	53.6
5′_Inx2_Right	5′-CACGACGTTCAGGAAATTCA-3′	53.0
Intron1_Inx2_Left	5′-TCGAGAAATTGCAGTGCATC-3′	53.5
Intron1_Inx2_Right	5′-GCGAAAAACCGACACACTTT-3′	54.3
Intron2_Inx2_Left	5′-CGTGCTTGAACCATCGTAGA-3′	54.9
Intron2_Inx2_Right	5′-GATTCAGAATGGCCAACGTC-3′	54,1
3′_Inx2_Left	5′-CCGTTATTTGCCAGCAGTTT-3′	54.0
3′_Inx2_Right	5′-GCCCAATGAAAGTTCCATCA-3′	53.3
Inx2_Exon1_Left	5′-ATGATCGTAAATTCGCTAAAACC-3′	51.6
Inx2_Exon1_Right	5′-AAACGGTCAACTTCGTGTGC-3′	56.3
Inx2_Exon2_Left	5′-TTTCCGGTCATTCATGGACT-3′	54.0
Inx2_Exon2_Right	5′-CTGGGGTGTTTTCAATTGTG-3′	52.2
Inx2_Exon3_Left	5′-TGATGAACGGTCGACAAATA-3′	51.5
Inx2_Exon3_Right	5′-GACAACTGTTGAATTCGTCCAT-3′	53.4
Inx2_Exon4_Left	5′-CGCCGATGTCATAACTTCAA-3′	52.9
Inx2_Exon4_Right	5′-CAGCACACGTATCGGAAAAC-3′	54.1
Inx2_Exon5_Left	5′-GAATTTGATTTGGCGCAGTT-3′	52.3
Inx2_Exon5_Right	5′-TTTGAACGCATACGGATTGA-3′	52.4

The protocol established by Siviero *et al*. [46] was used for sequencing. The BigDye Terminator sequencing kit (Applied Biosystem) and an automatic 16 capillary sequencer model ABI-3130 (Applied Biosystem) were used in collaboration with Prof. Dr Marie-Anne Van Sluys from Biosciences Institute (USP).

### 2.4. Sequence analyses

Analyses of the electropherograms were performed in a Linux operating system using the programs Phred, Phrap, CrossMatch and Consed 17, whose use licenses are free for academic purposes and obtained directly from the authors. The nucleotide sequences were analysed in the BlastX database. The alignments were performed using the ClustalX program (Multiple Sequence Alignment) [47] and the BioEdit program (Sequence Alignment Editor) [48]. To build the phylogenetic tree, the maximum-likelihood method available in the MEGA 6 program (Molecular Evolutionary Genetics Analysis) was used [49].

The ORF-Finder program was used to determine the amino acid sequence. To identify possible transmembrane domains, the TOPCONS program [50] was used. To build the topology of the Ra-Inx2 protein, the Protter program [51] was used and for the three-dimensional construction of the Ra-Inx2 protein prediction, the RaptorX program [52] was used. Other molecular biology tools were also used, such as the programs available on the National Center for Biotechnology Information (NCBI) website. The sequence of Ra-Inx2 was submitted to GenBank under accession no. MZ546417.

### 2.5. Immunofluorescence

To identify the location of the Ra-Inx2 protein in *R. americana* cells, immunofluorescence reactions were performed in the fat body and ovary. After dissecting the larvae, the tissues were transferred to a tube containing 3.7% formaldehyde for 15 min. Then, the tissues were washed twice in PBSA (140 mM NaCl, 2.7 mM KCl, 1.5 mM KH_2_PO_4_, 6.5 mM Na_2_HPO_4_), permeabilized with 1% Triton X-100 for 10 min and washed twice in PBSA. After, the primary antibody (anti-Innexin2 diluted to 1 : 100—kindly provided by Prof. Franka Eckardt of the University of Bonn in Germany; or anti-Innexin4 diluted to 1 : 100—kindly provided by Prof Liliach Gilboa of the Weizmann Institute of Science in Israel) was added and incubated at room temperature for 12 h. After this period, the tissues were washed twice with PBSA for 5 min and then the secondary antibody FITC (Sigma-Aldrich), diluted 1 : 200 in PBSA, was added and incubated for 2 h at room temperature. RNase 10 mg ml^−1^ was added and incubated for 1 h. The tissues were washed with PBSA and the slides were mounted with propidium iodide and Vecta Shield (Vector Laboratories, Burlingame, California, USA). The preparations were observed with the laser scanning confocal microscope (LSM510 - Zeiss), and the LSM Image Browser (Zeiss) was used to analyse the images.

### 2.6. *In situ* hybridization

The probe used for hybridization was synthesized from the genomic sequence, and primers Inx2_InSitu_Left and Inx2_InSitu_Right were used (table 1). The squashes of polytene chromosomes were prepared from the salivary gland of *R. americana* fixed in ethanol : acetic acid (3 : 1). Then, the chromosomes were denatured in 0.07 N NaOH for 5 min, then washed three times in 2 X SSC (3.0 M NaCl and 0.3 M sodium citrate pH 7.0), 70% ethanol and absolute ethanol. After drying, the slides received the hybridization mixture containing the probe previously marked with random primer digoxigenin-11-dUTP and denatured by heating. Hybridizations were performed at 58°C with 4 X SSC, for 12 h. After this period, chromosomal preparations were incubated with antibody anti-digoxigenin conjugated to fluorescein, and propidium iodide was used for counterstaining. The preparations were observed using the LSM510 laser scanning confocal microscope (Zeiss, Oberkochen, Germany) and the LSM Image Browser program was used to analyse the images.

### 2.7. Transmission electron microscopy

Tissues were dissected and fixed for 2 h with 2.5% glutaraldehyde and 2% formaldehyde in 0.1 M sodium cacodylate buffer pH 7.2. The fixed samples were washed in 0.1 M sodium cacodylate buffer pH 7.2 and post-fixed in 1% osmium tetroxide. The tissues were dehydrated in a graded ethanol and propylene oxide series. Resin infiltration was done with a 1 : 1 mixture of propylene oxide and EPON (Electron Microscopy Science, PA, USA) for 5 h, followed by pure Epon for 5 h. Next, the material was embedded in Epon and polymerized for 48 h. Semi-thin sections were cut using an ultramicrotome and stained with toluidine blue or haematoxylin/eosin. Appropriate regions of the ovaries and fat body were then thin sectioned at 70 and 90 nm and stained with 4% uranyl acetate and a 10% lead citrate solution. The material was analysed with a Jeol 1010 transmission electron microscope at 80 kV.

## 3. Results

### 3.1. Molecular characterization

**Figure 1.**
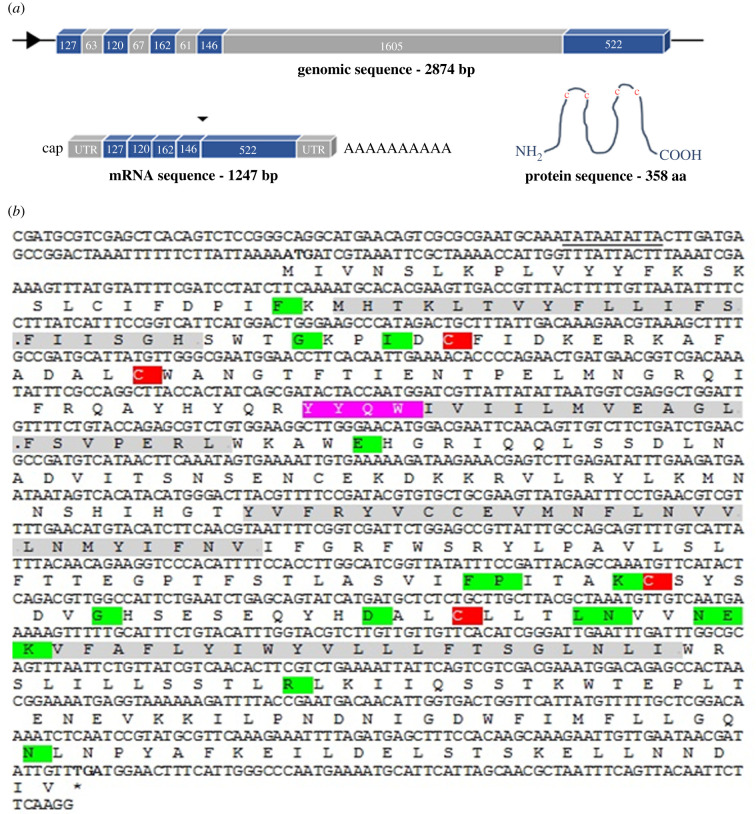
(*a*) Scheme showing the structure of Ra-Inx2, the genomic sequence (2874 bp), the mRNA sequence after being transcribed and processed (1247 bp) and the translated sequence of 358 aa. (*b*) Ra-Inx2 mRNA sequence with the consensus translation of the protein. The four transmembrane domains (TM1, TM2, TM3 and TM4) are highlighted in grey. At the beginning of the second transmembrane domain, the highly conserved amino acid sequence YYQW is highlighted in pink. The two cysteines located in the two extracellular loops (EL1 and EL2) are highlighted in red. Conserved amino acid residues are highlighted in green. The promoter region is underlined at the beginning of the sequence, and the start codon (ATG) and the stop codon (TGA) are highlighted in bold.

The genomic sequencing of Ra-Inx2 showed 2874 bp with five exons and four introns. The nucleotides conserved at the ends of the sequence of each intron (initial GT and terminal AG) were identified; these two pairs of nucleotides being important for the splicing machinery to recognize the sequence to be removed during mRNA processing for mature mRNA formation. The beginning of the sequence is characterized by four exons of approximately 120 to 160 bp and three introns of approximately 60 bp. However, the final part of the sequence is characterized by a long intron of 1606 bp and the last exon of 522 bp. A total ORF of 1077 bp codifying a putative protein of 358 amino acids represents the Ra-Inx2 (figure 1*a*).

To check whether alternative splicing occurs during mRNA processing in Ra-Inx2, the region of each exon was amplified from different tissues of *R. americana*, such as the fat body (third period of the fourth larval stage), salivary gland (third period), ovary (pupa), testis (second, third and fourth period) and embryo (first and fifth day of embryonic development). All the results obtained from Ra-Inx2 show no alternative splicing in the analysed tissues; all regions of exons are encoded.

**Figure 2.**
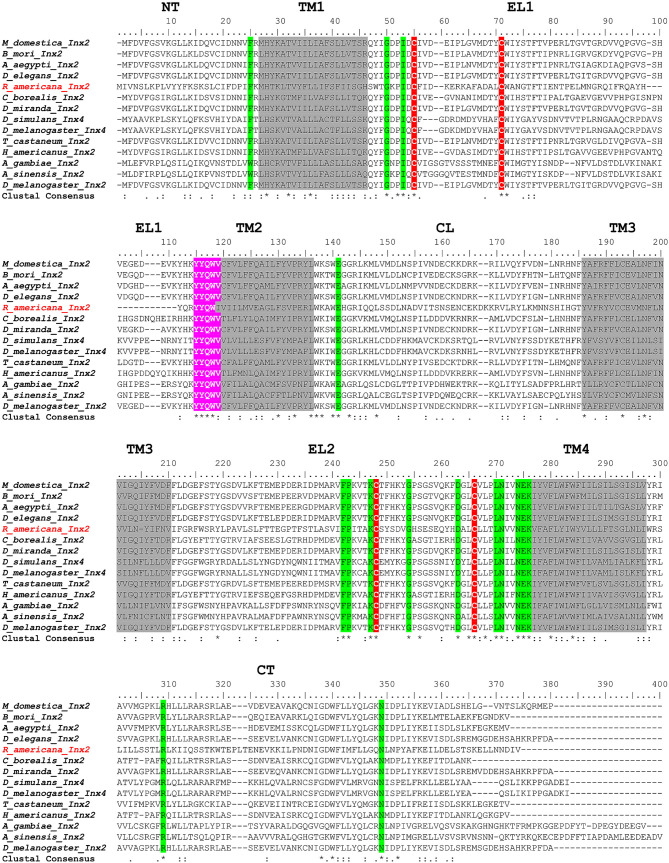
Alignment of the Ra-Inx2 protein consensus sequence and innexins of other species. NT represents the AT region; in grey, the four transmembrane domains (TM1, TM2, TM3 and TM4); EL1 and EL2 represent the regions of the extracellular loops, where the two red cysteines are located; CL represents the region of the intracellular loop; and CT represents the CT region. At the beginning of the second transmembrane domain, the highly conserved amino acid sequence YYQWV is highlighted in pink. Conserved amino acid residues are highlighted in green.

The putative Ra-Inx2 protein sequence has a conserved domain of 320 nucleotides in the superfamily of innexins. The characteristic regions of gap junction proteins were identified: four transmembrane domains in grey, two red cysteines located in the two extracellular loops and the conserved amino acid residues in green. The amino acid sequence YYQWV, highlighted in pink at the beginning of the second transmembrane domain (TM2), is highly conserved mainly among insects, being considered a signature among innexins (figure 1*b* and figure 2). However, the result we obtained in place of the last amino acid valine (V) was the amino acid isoleucine (I), but this variation has not yet been found in insects, only in sequences of innexins present in other invertebrates [4].

From the alignment of Ra-Inx2 with the innexin sequences of different species was identified the AT region, CT region, intracellular regions (CL), the hydrophobic regions were highlighted in grey corresponding to the four transmembrane domains (TM1, TM2, TM3 and TM4, respectively); the two extracellular loops (EL1 and EL2) with the two pairs of cysteines highlighted in red, and conserved amino acid residues among the innexins were highlighted in green (figure 2).

**Figure 3.**
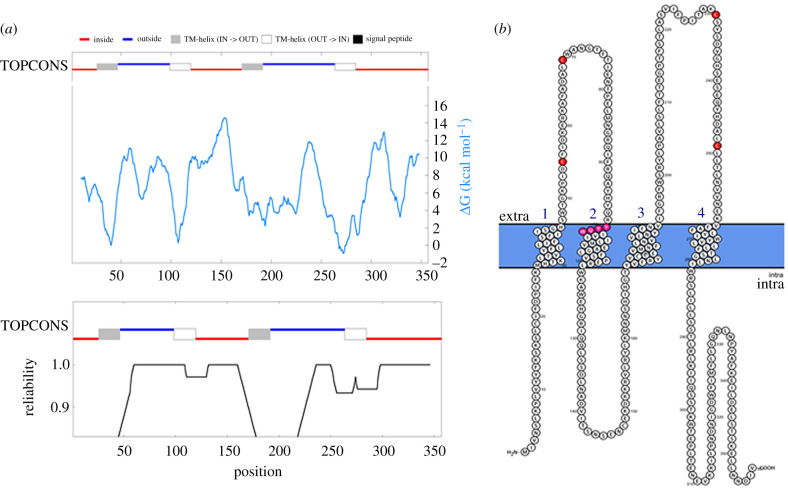
(*a*) Consensus prediction of membrane protein topology using TOPCONS. The intracellular regions are highlighted in red, and the extracellular regions are highlighted in blue. The four transmembrane domains are highlighted in grey and white boxes. (*b*) The topological structure of Ra-Inx2 using Protter. The four transmembrane domains are numerically highlighted in blue, the two pairs of cysteines located in the extracellular loops are highlighted in red and the conserved sequence YYQW is highlighted in pink at the beginning of the second transmembrane domain (TM2).

The putative protein sequence of Ra-Inx2 was analysed using the Topcons program to confirm the transmembrane structure. The *Δ*G value refers to the distance of each amino acid residue in the transmembrane. The graph highlighted the three intracellular regions in red (inside), corresponding to the amino-terminal region (NT), the intracellular loop (CL) and the CT region. In blue (outside), the two extracellular regions correspond to the two extracellular loops EL1 and EL2, respectively. The four transmembrane domains were also highlighted, with TM1 and TM3 in grey (TM-helix IN → OUT) and TM2 and TM4 in white (TM-helix OUT → IN) (figure 3*a*).

**Figure 4.**
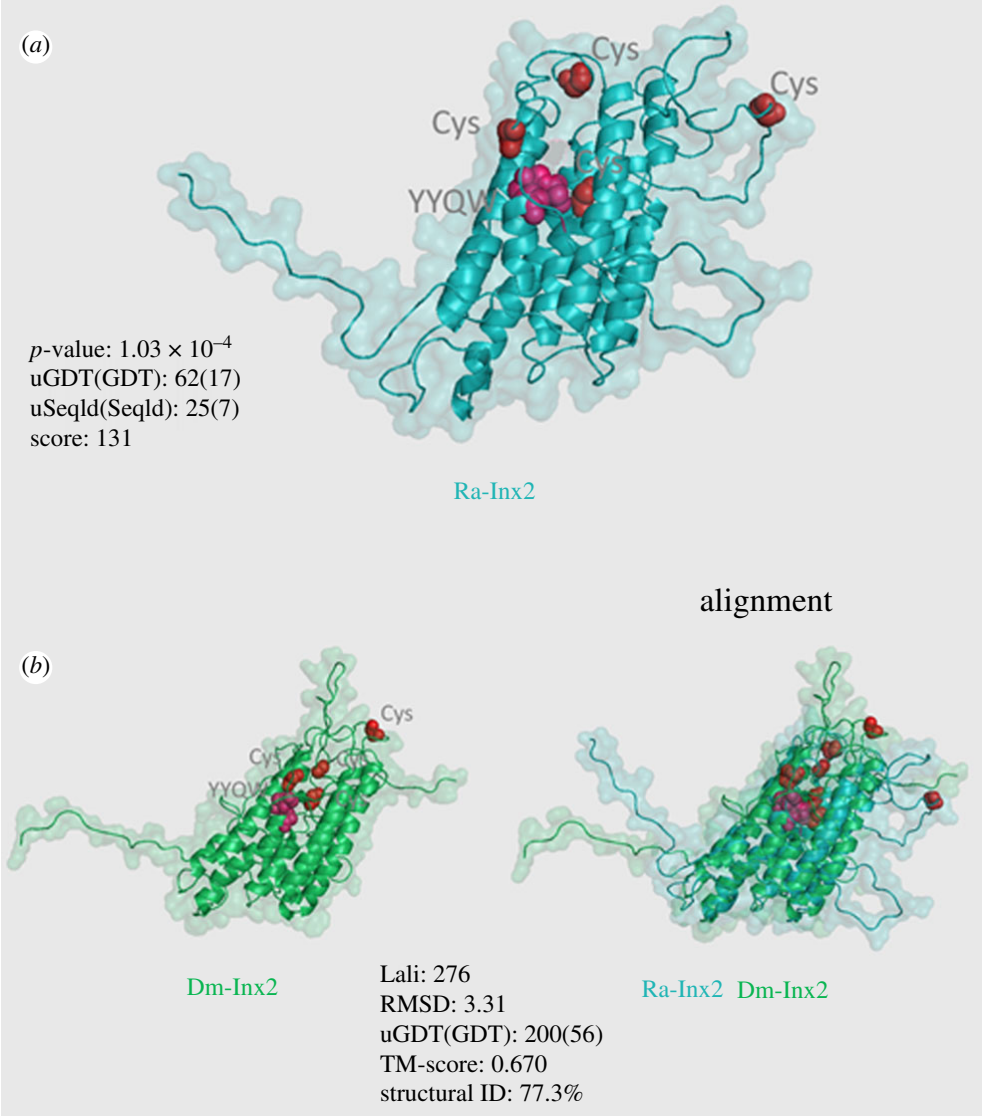
Prediction of the Ra-Inx2 protein using RaptorX. (*a*) *R. americana* Ra-Inx2 protein showing the conserved YYQW region in pink and the two pairs of cysteines (Cys) located in the two extracellular loops in red. (*b*) Alignment of Ra-Inx2 with the prediction *of D. melanogaster* protein Dm-Inx2.

The topological structure of the Ra-Inx2 protein was predicted from the putative amino acid sequence: the cysteine residues located in the extracellular loops are highlighted in red, and the conserved sequence YYQW is highlighted in pink. It was also possible to observe the alpha-helix domains corresponding to the TM transmembrane domains (figure 3*b*). The prediction of the three-dimensional structure of the Ra-Inx2 protein and alignment with the Dm-Inx2 protein of *D. melanogaster* was performed using RaptorX program (figure 4). The alignment showed 77.3% identity and TMscore of 0.670, confirming that the two proteins have similar folds. The structural ID was calculated based on the Lali value (length of alignment), showing the number of aligned amino acids.

Using the innexin sequences of other organisms available in the international NCBI database, we obtained Ra-Inx2 identity and similarity values. The term identity refers to the number of identical amino acids, and the term similarity refers to the number of amino acids with similar chemical properties. The values obtained were similar among different organisms, maintaining an average of approximately 34.1% identity and 55.1% similarity. Moreover, the organism that has a more similar sequence was of the species *Anopheles gambiae*.

**Figure 5.**
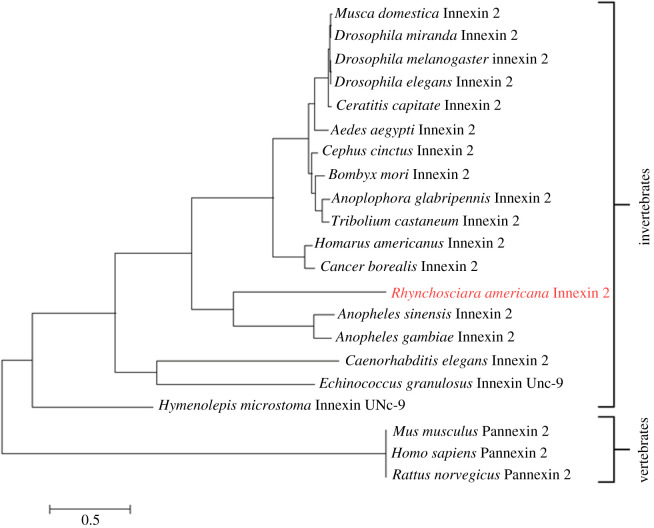
Phylogenetic analysis comparing the Ra-Inx2 (*Rhynchosciara americana* innexin2) protein sequence with the innexin sequences of other species. The tree obtained by the maximum-likelihood method. The evolutionary history was inferred using the JTT model. The data obtained were generated automatically based on the Neighbor-joining and BioNJ algorithms. The tree was drawn to scale, with branch lengths measured based on the number of replacements per site. The analysis involved 21 amino acid sequences.

Based on the amino acid sequence of Ra-Inx2, the MEGA 6 program [49] was used to construct a phylogenetic tree with the maximum-likelihood method. The evolutionary history was inferred using JTT model [53]. The data obtained were automatically generated based on the Neighbor-Join and BioNJ algorithms. The tree was drawn to scale, with the length of branches measured based on the number of substitutions per site. The analysis involved 21 amino acid sequences available from the international NCBI database (figure 5). Ra-Inx2 was grouped with the sequence of other arthropods, especially with dipterans of the Culicidae family, such as *A. gambiae* and *A. sinensis*. For the analyses, the innexin-2 sequences of different invertebrates and the sequences of the pannexin-2 proteins of vertebrates were used. Outgroups were separated in two ways: the first outgroup was invertebrates not belonging to the Arthropoda phylum, such as the nematode *Caenorhabditis elegans* and the flatworms *Echinococcus granulosus* and *Hymenolepis microstoma*. The second outgroup included vertebrates such as *Homo sapiens* and rodents *Mus musculus* and *Rattus norvegicus*.

### 3.2. Chromosomal location of Ra-Inx2 gene

**Figure 6.**
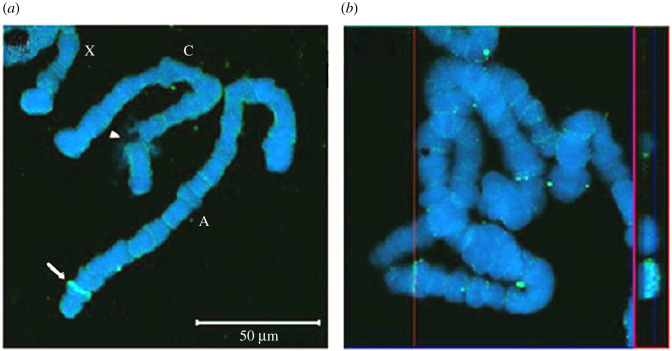
Laser scanning confocal microscope images. (*a*) *In situ* hybridization of the Ra-Inx2 gene (green—arrow) in salivary gland polytene chromosomes (DAPI-blue) of the *R. americana*. Chromosomes A, C and X are identified, and on the arrowhead indicates puff in region 3 of chromosome C. (*b*) Orthogonal section of chromosomes stack showing the *in situ* hybridization of the Ra-Inx2 gene in a band of the chromosome A 17 region.

The probe was synthesized from the genomic sequence, and the images were acquired using a laser scanning confocal microscope. *In situ* hybridization experiments showed that the Ra-Inx2 gene is in region 17 of chromosome A of *R. americana* salivary gland (figure 6*a,b*).

### 3.3. Expression of the Ra-Inx2 gene during larval development

**Figure 7.**
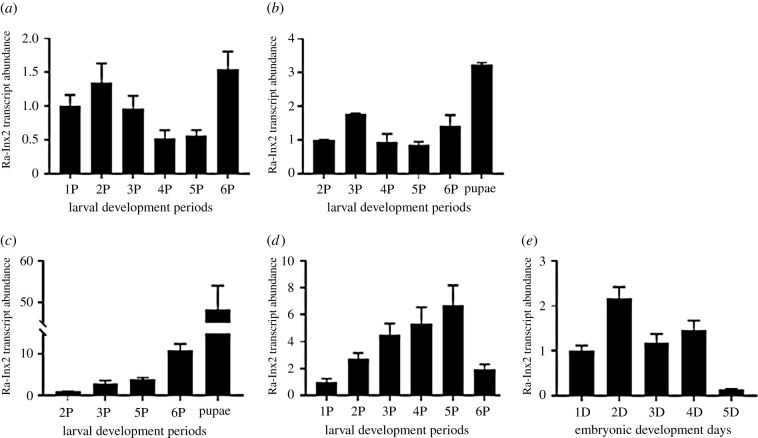
qPCR showing the expression of Ra-Inx2 in different tissues along with the development of *R. americana*. (*a*) Salivary gland, (*b*) fat body, (*c*) ovaries, and (*d*) testis during larval development and (*e*) embryos from different developmental days. The bars represent the standard error of the triplicate reactions.

Gene expression of Ra-Inx2 during the development of *R. americana* was analysed by using qPCR in the following tissues: salivary gland, fat body, ovary, testis and early development embryo. The salivary gland of *R. americana* is a tissue of large proportions in the larvae [2,54,55]. Physiologically, the salivary gland plays a fundamental role in larval development, actively participating in constructing the communal cocoon [56]. The analysis of Ra-Inx2 expression was evaluated from the first to sixth period of the fourth larval stage of development (figure 7*a*). In the sixth period, the Ra-Inx2 expression level was increased, being 1.5 times the level of the first period.

The fat body has a role in the intermediate metabolism in insects, participating in nutrition. In the larval stage, the cells have a flattened shape and are intimately connected to each other. At the end of the larval development, the fat body cells dissociate and reorganize in small clusters during the pupal stage [57]. During the fourth stage of larval development, the expression of Ra-Inx2 had a slight variation; an increased Ra-Inx2 expression was observed only at the beginning of the pupal phase, which coincides with tissue remodelling (figure 7*b*).

The *R. americana* ovary has characteristics different from the *Drosophila* in which the oocyte is connected by 15 nurse cells. In *R. americana*, all ovarian follicles develop synchronously and present only a single giant nurse cell attached to each oocyte. The ovarian follicle develops from the primordial germ cells that differentiate into germ stem cells after some mitotic divisions become germ cells. The last mitotic division of germ cells occurs early in the larval stage, giving rise to two cells with different destinations: the oocyte that enters in meiosis, while the nurse cell undergoes processes of polyploidy and polyteny [54]. In the ovary, Ra-Inx2 expression increased gradually during larval development. In the fifth period of larval development, the expression increased three times during the second period of the fourth stage. But it is in the pupal phase, a more significant increase in expression occurred; maximum Ra-Inx2 expression occurs on the eighth day of pupal development; 48.3 times compared with the second period of development (figure 7*c*).

Testis development is different from the ovary in which the oocyte chromosomes are arrested in meiosis and are little active transcriptionally since the transcriptional activity occurs in the nurse and in the FC. In the *R. americana* testis, the developmental process is more dynamic; the stage of growth of the last spermatogonia until the spermatocyte-I is quite long and begins in larvae of approximately 30 days of age. In the pre-meiotic stage, the chromosomes duplicate, and the RNA synthesis is very intense [58]. Ra-Inx2 gene expression in the testis increases gradually during larval development. The fifth period presented the highest level of expression, being 6.7 times compared with the second period. Soon after, in the sixth period, there was a sharp decrease in Ra-Inx2 expression level (figure 7*d*).

During its development, the embryo is composed of somatic and germinative cell lineages. The expression of Ra-Inx2 occurred at higher levels during the first 4 days of embryonic development. On the second day, the development was more expressive, being 2.1 times greater compared with the first day of development (figure 7*e*).

### 3.4. Ra-Inx2 immunofluorescence

**Figure 8.**
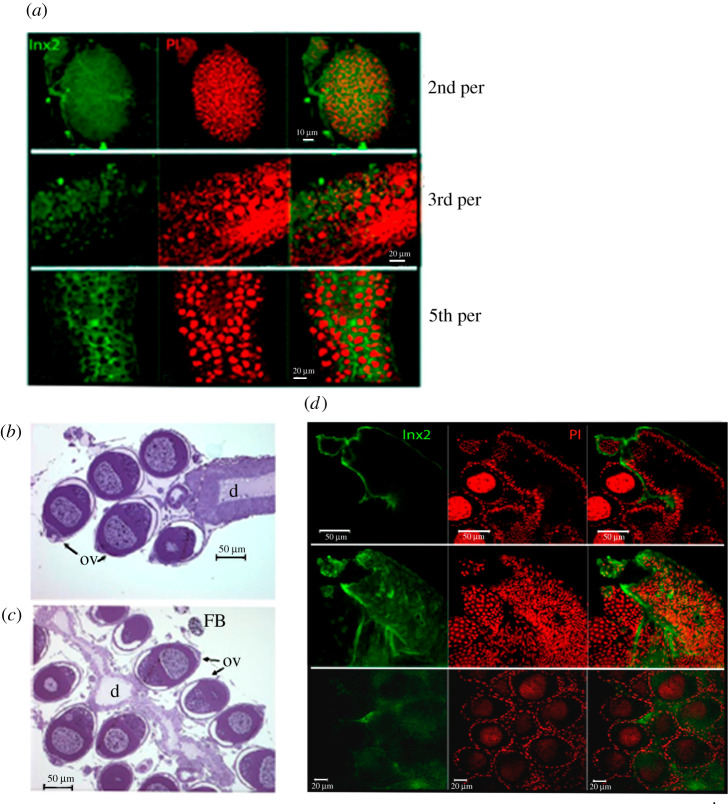
Ra-Inx 2 cellular localization: confocal laser scanning microscopy of total ovary preparation of *Rhynchosciara americana* larvae: (*a*) second and third period larvae and fifth period of the fourth larval stage. Channels separated in the first and second column and merged in the third. (*b*,*c*) Semi-thin sections of pupae ovary showing ovariole (ov), duct (d) and in (*c*) fatty bodies (FB). (*d*) Pupae ovary (6 days) showing optical slices 10, 20 and 30 of a stack of 70 acquired images in the *z*-axis, with separate fluorescence channels and merged. Ovary preparations were subjected to immunofluorescence for innexin-2 (FITC-green). DNA stained with propidium iodide (PI).

The immunofluorescence reaction with the anti-innexin2 antibody resulted in diffuse labelling on the surface of the ovary of young larvae, suggesting that Ra-Inx2 was in FC. In addition, the positivity (green) was more intense in the fifth period, among the ovarian follicles, similarly to the labelling observed in the third period of the fourth larval stage, localization suggestive of somatic cells. (figure 8*a*).

The labelling observed in extra-FC would explain the fact that older pupae present high expression of Ra-Inx2 mRNA, which at first was intriguing because the nurse cell, responsible for RNA synthesis in the oocyte at this age, begins to enter a process of regression and death, and the Ra-Inx2 labelling was detected in FC at this stage. In agreement with these data, intense fluorescence was observed in cells of a tubular structure in the ovary, as illustrated in figure 8*b–d*.

The organization of the ovarioles around a duct-like structure was evidenced by several techniques, from histological sections to three-dimensional reconstruction of the ovary from images obtained by light-sheet microscopy. Figure 8*b*, *c* illustrates the organization of the *R. americana* ovary observed in semi-thin sections of transmission electron microscopy preparations. The ovarian follicles are distributed around the duct, and the FC are continuous with the ductal cells. In addition, among the follicles, there are also stromal cells. These cell types are all somatic origin, and data suggest a commitment of these cells to the Ra-Inx2 expression.

**Figure 9.**
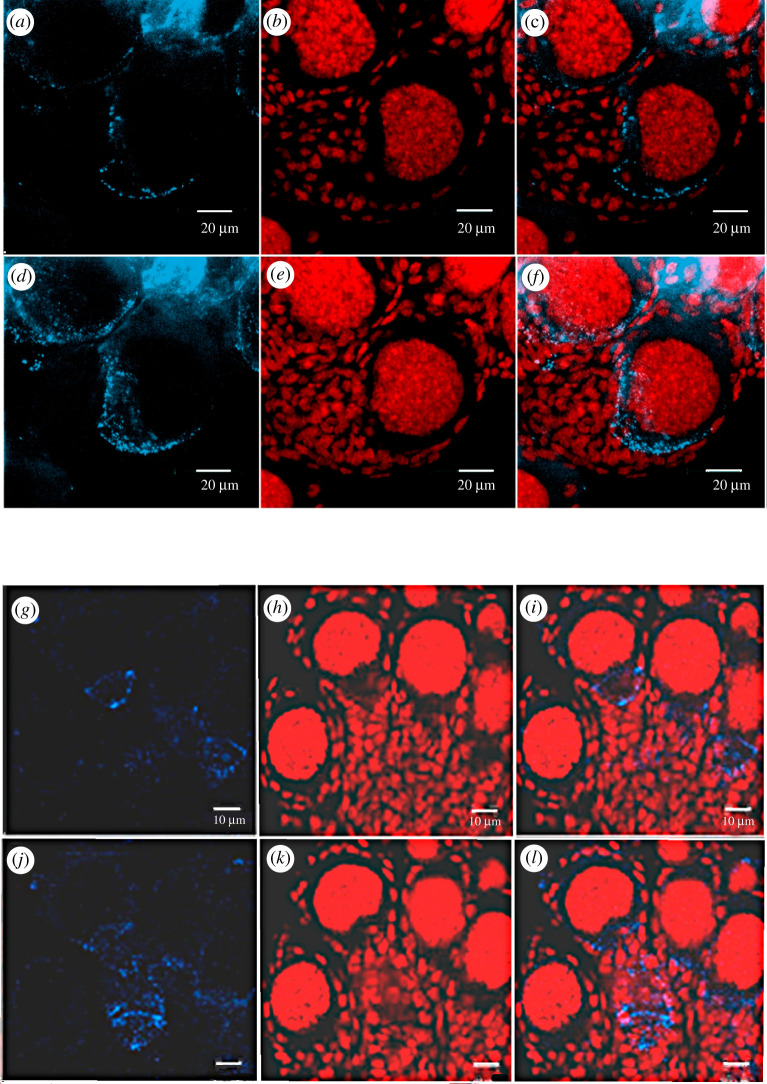
Ra-Inx 4. Immunolocalization of Ra-Inx4 in the ovary of the third period of the fourth larval stage (*a*–*f*) and pupae (*g*–*l*): DNA was stained using propidium iodide (red), Ra-Inx4 revealed with secondary Ab-CY5 (blue) and third column, merged channels.

Anti-innexin2 antibody reaction in the *Rhynchosciara americana* ovary preparations did not allow structures resembling gap junctions to be visualized, so the ovaries were analysed for Ra-Inx4 immunofluorescence to compare with Ra-Inx2 result. The immunolocalization data of Ra-Inx4 within the ovariole suggest its association with the germline, different from Ra-Inx2 localization (figure 9). A similar Ra-Inx4 location was observed both in the larval and pupal stages. Therefore, this protein must be associated with cell communication from the beginning of the pupal stage, thinking about the innexin canonical function. However, it cannot leave aside the hypothesis of the participation of innexins in other cellular processes, similar to what happens with connexins.

The fat body is an organ that has ecdysone-dependent remodelling in the larva/pupa period, as recently described by the group [57], which makes it a relevant study target. Therefore, the Ra-Inx2 and Ra-Inx4 expression were determined in the fat body during the final stage of larval development. Innexins should play some role in the biology of this tissue when it looks like sheets of epithelial-like cells and not in the more advanced stages of development, when it becomes small cell aggregates, agreeing with the remodelling that occurs at the beginning of the pupal stage. Ra-Inx 2 seems to be most relevant in the fat body at the end of larval life, keeping its expression at levels practically equal to the second period of larval development, decreasing drastically in the 1-day-old pupa.

**Figure 10.**
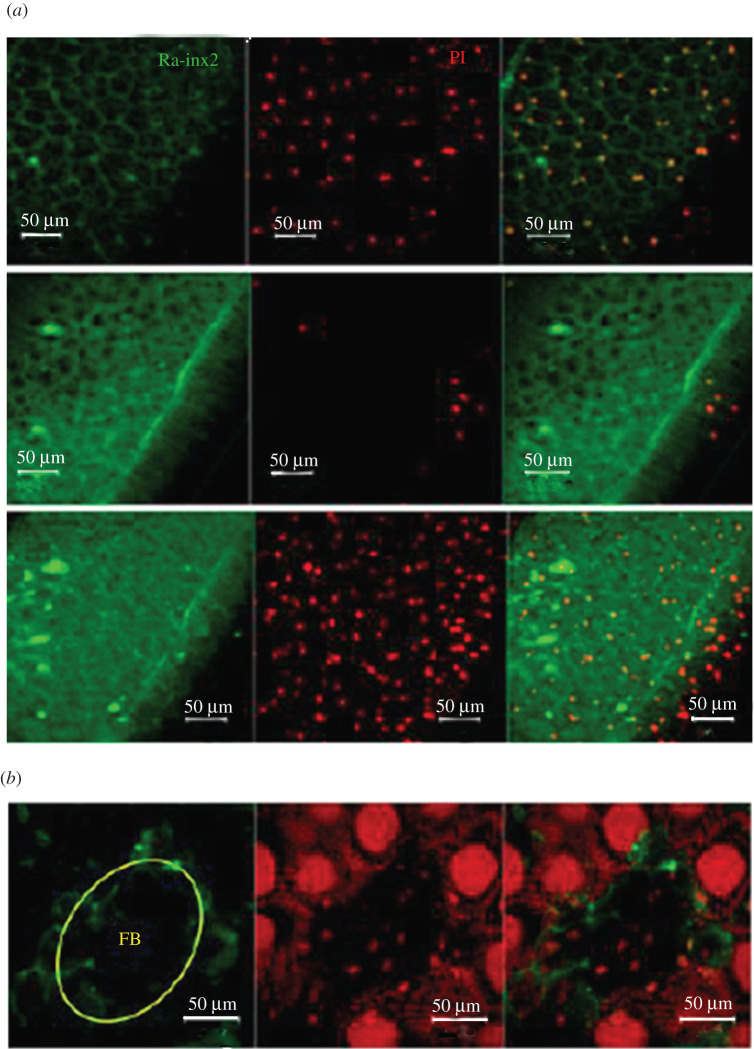
Ra-Inx2 immunolocalization in the fat body of the fifth period of the fourth larval stage observed in different optical sections under the confocal laser scanning microscope, showing the labelled pattern most superficial and intermediates in the tissue. In (*b*), fat body cells are marked in a yellow circle in the 6-day-old pupae ovary. Immunofluorescence with anti-innexin-2 antibody (green) and DNA stained with propidium iodide (red).

The immunolocalization of innexin2 in the larval fat body (fifth period of the fourth stage) shows positivity at the cellular boundaries and is more evident at the base of the cells (figure 10). Interestingly, in the 6-day pupae (figure 10*b*), fat body cells are negative to Ra-Inx2.

### 3.5. Transmission electron microscopy

The evidence of the location of gap junctions by immunofluorescence observed under confocal laser scanning microscope pointed to the relevance of visualizing structures corresponding to these types of junctions, under the transmission electron microscope. Previous data focusing on the development of the *R. americana* ovary, both from Basile in the 1970s and more recently of the group, had a greater concern with the ovariole and the relation between the nurse cell and oocyte. Revisiting these data and making a broader morphological study now, the existence of other cells in the organs that also express innexins and have gap junctions, is evident. Importantly, mRNA expression profiles relative to whole organs, containing material from different cell types. In the specific case of ovaries and testes, there is a mixture of somatic and germinative cells, reinforcing the importance of new morphological studies.

**Figure 11.**
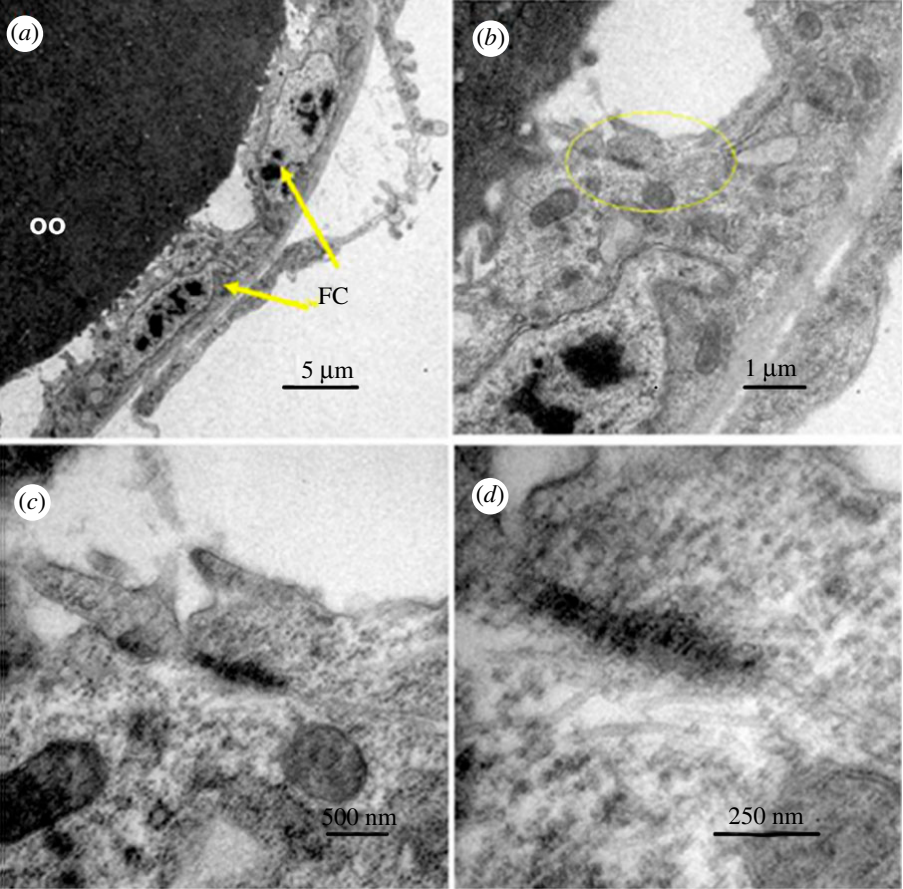
Junctions between 2-day-old pupae ovary cells. Transmission electron microscope images showing detail of FC surrounding the oocyte (oo) (*a*) lower magnification; (*b*) higher magnification with the junction region highlighted in yellow; (*c*,*d*) progressive magnifications in the same region.

**Figure 12.**
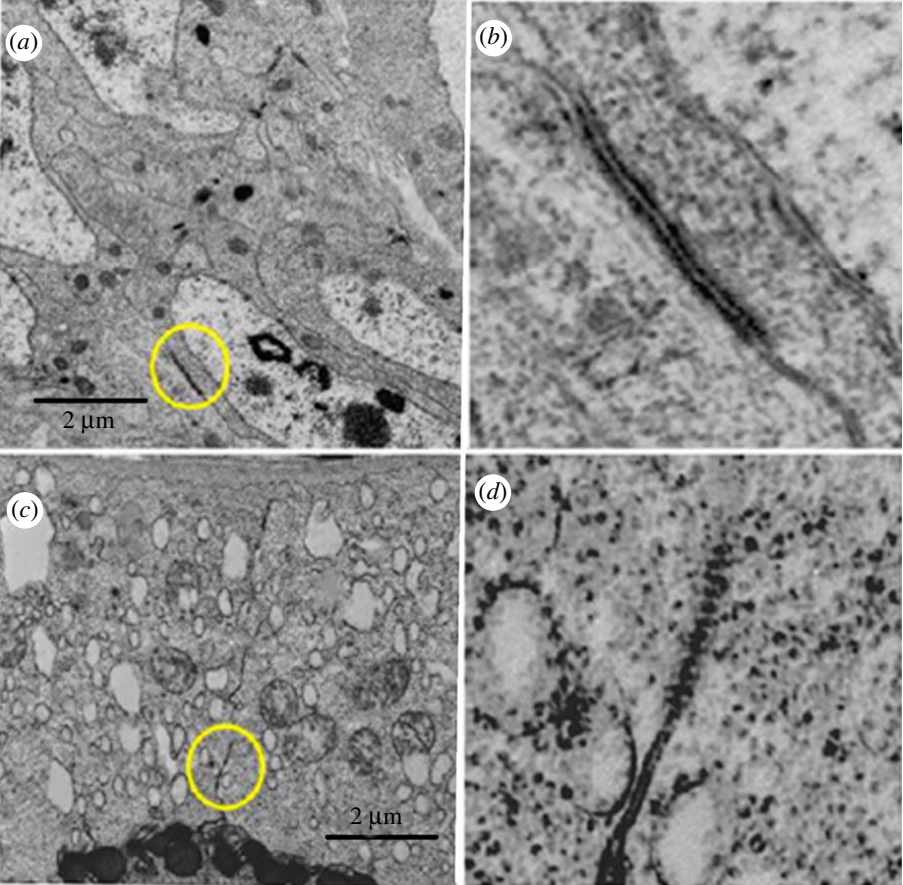
Six-day-old pupae ovary. Images obtained under the transmission electron microscope: (*a*) cells of the base of the ovarioles with junction marked in yellow and shown in higher magnification in (*b*,*c*) follicular cells with junction marked in yellow. (*d*) Greater magnification of the junctions.

Cell junctions like gap junctions were observed between somatic cells of the ovaries, mainly between FC, but also between cells at the base of the ovarioles that may correspond to ductal cells. Figures 11 and 12 show images of young pupae ovariole (2 days old) and of 6-day-old pupae, respectively, obtained under the transmission electron microscope. More prominent junctions were observed between FC in the pupal stage. Comparing the images of 2-day and 6-day pupae (figures 11 and 12), those shown in figure 11 look like smooth septate junctions. In figure 12, between the cells at the base of the ovarioles, it looks like a gap junction, whereas in FC, the gap junction continues in a smooth septum.

**Figure 13.**
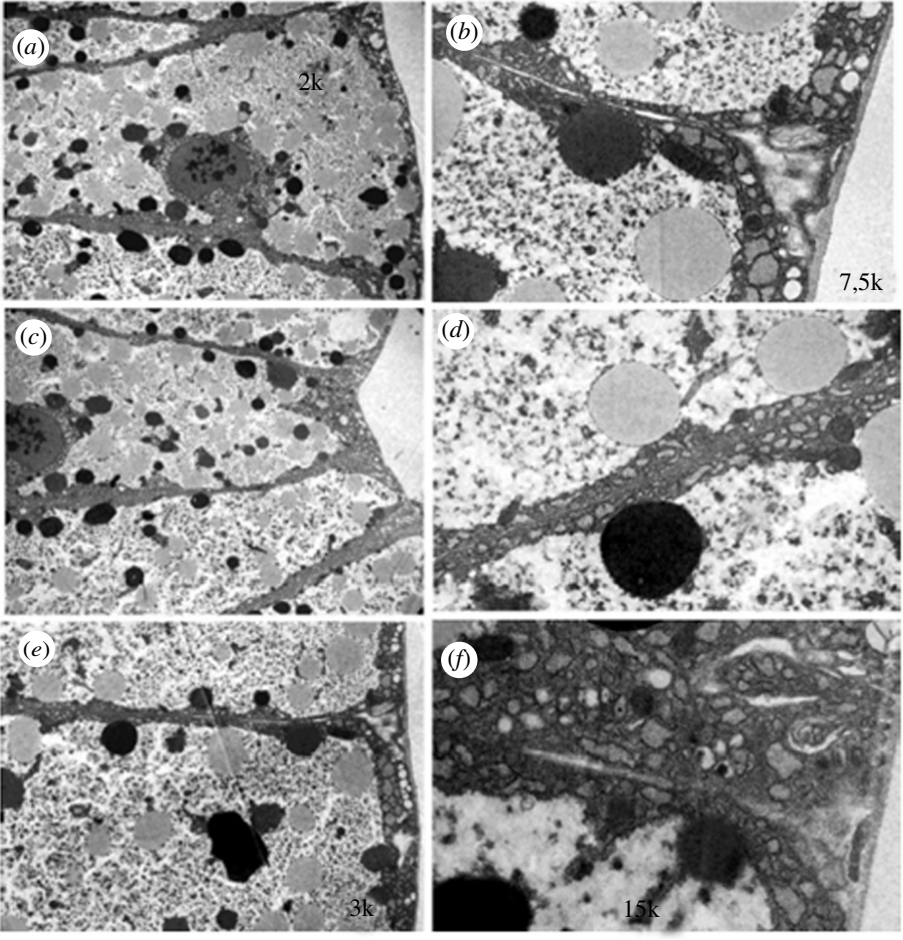
Fat body of larvae in the second period of the fourth larval stage. Images obtained by transmission electron microscope of border regions between cells (*a,c,e*) and some details in greater magnification in (*b*,*d*,*f*). The granular endoplasmic reticulum is displaced towards the plasmatic membrane making it challenging to visualize the junctions.

**Figure 14.**
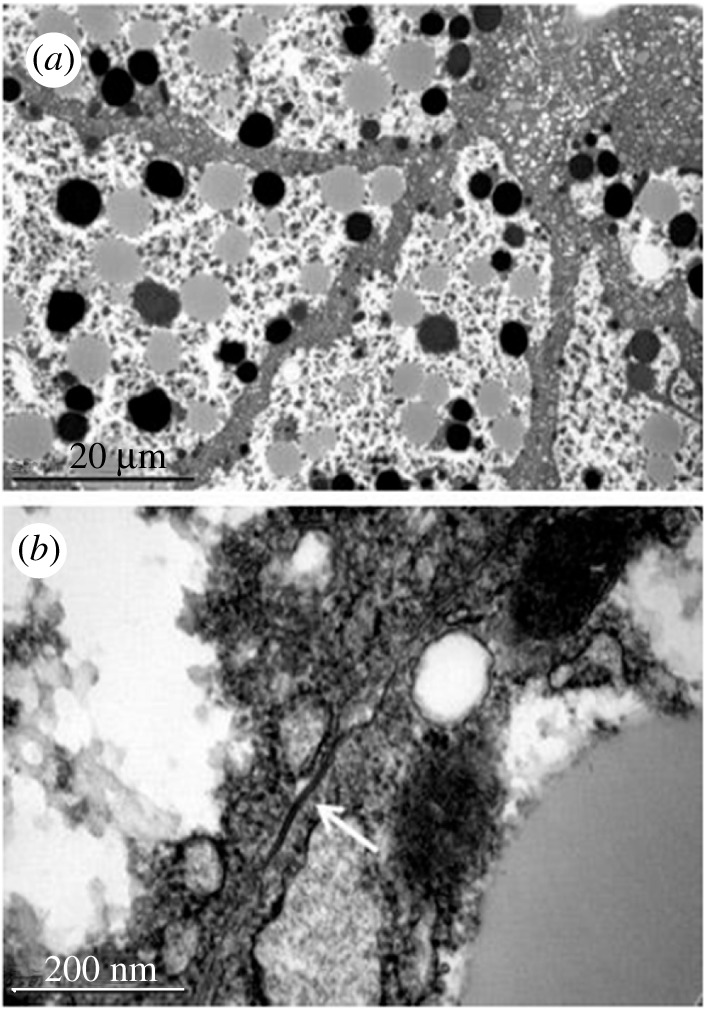
Fatty body of larvae in the third period of the fourth larval stage. Transmission electron microscope images of boundary regions between cells, at + lower magnification in (*a*) and in detail in (*b*), showing a region with junction.

In the fat body of young larvae, the granular endoplasmic reticulum is displaced to the cell boundaries, which makes it challenging to observe junctions (figure 13). These could be observed in older larvae, where protein synthesis would be less active. Figure 14 illustrates the morphology of these cells at lower magnification and a border region between the cells, showing a gap junction.

## 4. Discussion

The Ra-Inx2 genomic DNA sequence analysis resulted in 2874 base pairs, with the presence of four intronic regions corresponding to 1797 base pairs, or 62.5% of the sequence. The search for alternative splicing in different *R. americana* tissues was performed, amplifying by PCR the five exon regions and analysing the data, it was possible to conclude that there is no alternative splicing in the Ra-Inx2 gene. Stebbings *et al*. [59] show that Dm-Inx2 splicing occurs in a single intron located outside the ORF, then splicing does not cause changes in the predicted polypeptide sequence. However, Crompton *et al*. [60] showed that Dm-Inx8 splicing affects the ORF, and consequently, there are changes in the polypeptide sequence, these variations occurring differently in each tissue. More recently, Calkins *et al*. [61] showed that in *Ae. aegypti* alternative splicing occurs in Ae-Inx1 and Ae-Inx3, affecting the CT end of these two innexins. Calkins *et al*. [61] also showed that Ae-Inx2 has two exons encoding a protein of 359 amino acids and Ae-Inx7 four exons encoding a protein of 407 amino acids, and these two innexins did not identify alternative splicing.

The Ra-Inx2 mRNA encodes a putative protein sequence of 358 amino acids, in agreement with the sequence of innexins characterized in other organisms. The identity of Ra-Inx2 as a gap junction transmembrane protein was confirmed based on the conserved amino acid residues, the two cysteines located in the extracellular loops and the sequences that correspond to the four transmembrane domains. The cysteine residues located in the extracellular loops are essential for anchoring the canal in adjacent cells [62]. In vertebrates, each extracellular loop has three highly conserved cysteine residues, whereas in invertebrates, innexins have up to two cysteine residues, the exception being innexin-4 which has the third cysteine residue in the EL1 and EL2 loops [63]. Another characteristic found is the sequence of the first four amino acids of the YYQWV sequence, specifically located at the beginning of the second transmembrane domain (TM2), the function of this sequence is still unknown, however, it is considered a signature among the innexins [5,64]. The second transmembrane domain has a proline, which is also part of the identity of innexins.

Interestingly, this amino acid is also conserved in the second transmembrane domain of connexins and is associated with the activation of conformational changes in the protein [65,66]. The alignment showed that the transmembrane domains have an identical size between the different organisms, with TM1, TM2, TM3 and TM4 having, respectively, 20, 22, 25 and 21 amino acids. The two extracellular loops EL1 and EL2 have 52 and 66 amino acids, respectively. According to Bauer *et al*. [11] in *D. melanogaster* the EL1 and EL2 loops of Dm-Inx2 have 62 and 63 amino acids, respectively. The results obtained from the analysis of the sequence are in accordance with the results acquired from the TOPCONS program, and in the graph, the value of *Δ*G represents the distance of each amino acid residue in relation to the membrane [67]. Confirming that the amino-terminal (NT) and CT ends are in the cytoplasmic region. Studies have associated that the NT and CT regions have a regulatory role in the channel and act in the formation of communicating junction plaques [68,69]. The Ra-Inx2 alpha-helix domains were identified using the RaptorX program. As found in other junction proteins, such as claudins and occludins, the alpha-helix domains of connexins and innexins correspond to the regions where the transmembrane domains are present [9]. The prediction of the three-dimensional structure of the Ra-Inx2 protein shows an identity of 77% concerning Dm-Inx2 and a TMscore of 0.670, confirming that the two proteins have similar folds.

Based on the sequence of amino acids, a phylogenetic tree was constructed comparing the sequence of innexin-2 from other organisms available on GenBank. The tree was built in the MEGA 6 program using the maximum-likelihood method with a bootstrap of 1000 replications, which calculates the percentage of occurrence reliability for a given node in the tree as a percentage. As expected, Ra-Inx2 was grouped with other arthropods, mainly with the dipterans of the Culicidae family, such as *A. gambiae* and *A. sinensis*, proving the values obtained for identity and similarity. Arthropods represent the largest phylum in the animal kingdom, representing approximately 85% of the described animals. The subphylum Crustacea, represented in the tree by *Homarus americanus* and *Cancer borealis,* is placed in the same group as insects, confirming the level of conservation among arthropods. Recent studies have confirmed that insects are positioned phylogenetically in the same group as crustaceans [22,43,70]. In general, the results acquired are in accordance with the same phylogenetic pattern presented in the literature [4,71].

Gene expression indicated that a higher expression of Ra-Inx2 occurs in the salivary gland in the sixth period of the development of *R. americana.* In parallel during this period, apoptosis occurs in the salivary gland cells occurring during histolysis of this tissue [57]. However, the overexpression of Sl-Inx2 and Sl-Inx3 in *Spodoptera litura* cells and ectopic expression in *Spodoptera frugiperda* cells promoted cell apoptosis [72]. Connexins have also been associated with the cell death process. For example, the silencing of connexin-32 slows down the process of cell death [73]. In the fat body, the increased expression of Ra-Inx2 occurred in the pupal stage. According to Brandão *et al*. [57] the levels of the hormone 20-hydroxyecdysone have a peak in the pupal phase, and in parallel, the fat body cells undergo a reorganization process. The process of fat body remodelling in *R. americana* comprised a discrete detection of cell death by TUNEL assay characteristic of the apoptosis process. Active hemichannels may be involved with cell death signalling pathways or inhibiting cell survival pathways [43]. The activation of the channel could increase the amount of intracellular Ca^2+^ increasing the mitochondrial outer membrane permeabilization (MOMP). MOMP is a factor that induces apoptosis, causing the release of cytochrome C and activation of the caspase pathway [43]. The Ra-Inx2 expression may be involved during metamorphosis in *R. americana*, possibly participating in the remodelling of the fat body and the histolysis of the salivary gland.

Ra-Inx2 expression increased throughout the fourth stage of development in the ovary, which may be associated with the fact that the ovarian follicle increases considerably in size from the third period. During this period, the nurse cell goes through endo-replicative cycles, occurring in the polyploidy process [54]. However, the highest levels of Ra-Inx2 expression occur in the ovary in the pupal phase; in this period, the nurse cell goes through the process of polyteny, having its potential for transcription increased, while the oocyte is stopped in meiosis, with little transcriptional activity. Nurse and FC are responsible for the intense synthesis of mRNA. Stebbings *et al*. [63] showed the expression in the *D. melanogaster* ovary of Dm-Inx1, Dm-Inx2, Dm-Inx3, Dm-Inx4 and Dm-Inx7. Other studies show that Dm-Inx2 was co-localized with Dm-Inx3 in somatic cells and co-localized with Dm-Inx4 in a germ cell line [40]. Dm-Inx2 is expressed in somatic and germ cells, as well as Ra-Inx2. The RNAi knockdown of Inx1 and Inx3 in *Drosophila* ovarian follicles resulted in follicles without ovaries, small ovaries and few follicles [74]. Recent studies in *D. melanogaster* ovaries demonstrate that Inx2 and Inx3 act on border cells regulating microtubules, with Inx4 having a similar role in the oocyte. The RNAi knockdown of these innexins destabilizes the microtubules causing morphological changes in the border cells and oocyte [75]. In the testis of *R. americana*, Ra-Inx2 showed an expression pattern found in ovaries, and this pattern was also identified by Hong *et al*. [20] in *B. mori*. The expression of Bm-Inx2, Bm-Inx3 and Bm-Inx4 in the testis was very similar to the expression found in the ovary during the larval, pre-pupal and pupal phase, with Bm-Inx4 being the most expressed innexin [20,21]. In *Ae. aegypti*, the expression of Ae-Inx2 was considered strong in the testis [61]. In the gonads, the expression of Ra-Inx2 was observed throughout the development of the ovary and testis, indicating that this innexin can participate in oogenesis and spermatogenesis. During embryogenesis, the expression of Ra-Inx2 also occurred, and during embryonic development, cellular communication is essential. Therefore, Ra-Inx2 was consistently expressed in all the tissues studied throughout the development of *R. americana*. In the embryo, the expression of Ra-Inx2 remained constant throughout the 1–4 days of development; this expression profile was also observed by Hong *et al*. [20] in *B. mori*, and the expression of Bm- Inx2 and Bm-Inx3 maintained the same expression profile throughout embryonic development, different from Bm-Inx4 that was expressed only on the first day of development [20,21]. In *D. melanogaster*, the innexins Dm-Inx1, Dm-Inx2 and Dm-Inx3 participate in the dorsal development of the embryo [27]. Holcroft *et al*. [37] demonstrated that Dm-Inx1 and Dm-Inx2 participate in the development of the central nervous system in glial cells. It has also been shown that Dm-Inx2 participates in the organization of epithelial tissue during embryogenesis [31,34].

The Ra-Inx2 gene is in region 17 of chromosome A by *in situ* hybridization, a somatic chromosome, differently from *Drosophila*. Dm-Inx2 gene is located on X chromosome of *D. melanogaster* in 6E4 region, which includes a cluster of innexins genes, such as Dm-Inx1 and Dm-Inx7 [63]. To show the localization of the Ra-Inx2 protein, immunolocalization was performed on the ovary during the fifth period of development, showing that Ra-Inx2 is preferentially located in somatic cells of the ovary. In the *D. melanogaster* ovary, the location of Dm-Inx2 was also preferentially located in somatic cells. According to Bohrmann & Zimmermann [32], the Dm-Inx2 innexin is in the apico-lateral region of the FC, and it can interact with Dm-Inx4 in the oolema germline cells. Stebbings *et al*. [63] show that Dm-Inx2 can form homotypic channels or can co-localize with Dm-Inx3 forming heterotypic channels.

Take all together, gap junction channels are fundamental for the physiology of multicellular organisms. In particular, innexin-2 has become one of the most studied innexins among different organisms due to its characteristic of acting in different tissues of somatic and germinative lineages.

## Data accessibility

This article has no additional data.
